# Supplementary material for: Connecting MHC-I-binding motifs with HLA alleles via deep learning
Source: Commun Biol. 2021 Oct 18;4:1194. doi: 10.1038/s42003-021-02716-8 (PMC8523706; doi:10.1038/s42003-021-02716-8)
Supplement: Supplementary file 1 — Supplementary Information [file 42003_2021_2716_MOESM1_ESM.pdf]

# Supplementary Information for: Connecting MHC-I-binding motifs with HLA alleles via deep learning

Ko-Han Lee[1], Yu-Chuan Chang[1], Ting-Fu Chen[1], Hsueh-Fen Juan[1,2,3,4], Huai-Kuang Tsai[1,5], Chien-Yu Chen[1,6*]

[1]Taiwan AI Labs, Taipei 10351, Taiwan;

[2]Graduate Institute of Biomedical Electronics and Bioinformatics, National Taiwan University, Taipei 10617, Taiwan;

[3]Department of Life Science, National Taiwan University, Taipei 10617, Taiwan;

[4]Center for Computational and Systems Biology, National Taiwan University, Taipei 10617, Taiwan;

[5]Institute of Information Science, Academia Sinica, Taipei 11529, Taiwan;

[6]Department of Biomechatronics Engineering, National Taiwan University, Taipei 10617, Taiwan;

*Address correspondence to: chienyuchen@ntu.edu.tw (C-Y. C.)

**Supplementary Table 1. Summary of the datasets used in training, validating, and evaluating the MHCfovea's predictor**

| | D-E ratio | Binding Assay | | Ligand Elution | Protein Decoy | Random Decoy |
|---|---|---|---|---|---|---|
| | | Positive | Negative | | | |
| Training | 30 | 38,793 | 110,209 | 226,800 | 1,068,270 | 5,801,583 |
| | 60 | | | | 2,136,540 | 11,603,163 |
| | 90 | | | | 3,204,791 | 17,404,743 |
| Validation | 30 | 2,088 | 5,754 | 11,937 | 174,361 | 179,055 |
| Benchmark | 30 | 0 | 0 | 127,371 | 1,980,947 | 2,036,733 |

**Supplementary Table 2. The AP score of the ensemble model trained under different D-E ratios in the overall training dataset against D-E ratios in the downsized dataset**

| AP | | D-E ratio in the overall training dataset | | |
|---|---|---|---|---|
| | | 30 | 60 | 90 |
| D-E ratio in the downsized dataset | 1 | 0.877 | 0.879 | 0.879 |
| | 5 | 0.895 | 0.897 | 0.898 |
| | 10 | 0.889 | 0.893 | 0.895 |
| | 15 | 0.882 | 0.887 | 0.889 |
| | 30 | 0.861 | 0.871 | 0.875 |

**Supplementary Table 3. The AUC score of the ensemble model trained under different D-E ratios in the overall training dataset against D-E ratios in the downsized dataset**

| AUC | | D-E ratio in the overall training dataset | | |
|---|---|---|---|---|
| | | 30 | 60 | 90 |
| D-E ratio in the downsized dataset | 1 | 0.989 | 0.989 | 0.989 |
| | 5 | 0.990 | 0.991 | 0.991 |
| | 10 | 0.989 | 0.990 | 0.990 |
| | 15 | 0.988 | 0.989 | 0.989 |
| | 30 | 0.984 | 0.985 | 0.985 |

**Supplementary Table 4. Summary of the training dataset used in each predictor**

| | MHCfovea | NetMHCpan4.1 | MHCflurry2.0 | MixMHCpred2.1 |
|---|---|---|---|---|
| # of alleles | 150 | 161 | 174 | 67 |
| # of experimental measurements | 375,802 | 722,304 | 522,132 | 252,165 |
| # of decoys | 20,609,534 (90x) | 10,118,592 | 33,178,365 | 0 |
| | 6,869,853 (30x) | | | |

30x and 90x represent the D-E ratio of the overall training dataset

**Supplementary Table 5. Performance on the benchmark dataset**

| Predictor | AUC | AUC0.1 | AP | PPV |
|---|---|---|---|---|
| MHCfovea (90x) | 0.977 | 0.892 | 0.841 | 0.789 |
| MHCfovea (30x) | 0.977 | 0.892 | 0.832 | 0.780 |
| NetMHCpan4.1 | 0.958 | 0.859 | 0.825 | 0.783 |
| MHCflurry2.0 | 0.96 | 0.825 | 0.74 | 0.71 |
| MixMHCpred2.1 | 0.942 | 0.823 | 0.767 | 0.723 |

30x and 90x represent the D-E ratio of the overall training dataset
AUC0.1: AUC with a restriction of the false positive rate under 0.1

**Supplementary Table 6. List of the 16 unobserved alleles in the benchmark dataset**

| Alleles | Unobserved for MHCfovea | Unobserved for all compared predictors |
|---|---|---|
| A*24:07 | V | V |
| A*33:03 | V | |
| A*34:01 | V | V |
| A*34:02 | V | V |
| A*36:01 | V | V |
| B*07:04 | V | V |
| B*15:10 | V | |
| B*35:07 | V | |
| B*38:02 | V | V |
| B*40:06 | V | V |
| B*55:01 | V | |
| B*55:02 | V | |
| C*03:02 | V | V |
| C*04:03 | V | V |
| C*08:01 | V | |
| C*14:03 | V | V |

**Supplementary Table 7. Summary of the four groups in the benchmark**

| Allele tag | Peptide tag | # of Experimental measurements | # of Decoys |
|---|---|---|---|
| Observed | Similar | 44,149 | 1,667,879 |
| Observed | Dissimilar | 52,044 | 1,363,577 |
| Unobserved | Similar | 14,080 | 540,608 |
| Unobserved | Dissimilar | 17,098 | 445,616 |

**Supplementary Table 8. List of the multi-cluster HLA groups**

| | | | | | | | | |
|---|---|---|---|---|---|---|---|---|
| A*30 | A*34 | A*66 | A*68 | A*69 | A*80 | B*13 | B*14 | B*15 |
| B*35 | B*37 | B*38 | B*40 | B*41 | B*44 | B*46 | B*49 | B*53 |
| B*56 | B*67 | B*78 | C*03 | C*04 | C*07 | C*14 | C*15 | C*16 |

**Supplementary Table 9. Results of the hyperparameter optimization (validation dataset)**

| batch size | learning rate | AUC | AUC0.1 | AP | PPV |
|---|---|---|---|---|---|
| 16 | $10^{-5}$ | 0.980 | 0.873 | 0.810 | 0.751 |
| 16 | $10^{-4}$ | 0.981 | 0.870 | 0.805 | 0.744 |
| 16 | $10^{-3}$ | 0.974 | 0.852 | 0.783 | 0.733 |
| 32 | $10^{-5}$ | 0.979 | 0.865 | 0.799 | 0.740 |
| 32 | $10^{-4}$ | 0.981 | 0.874 | 0.810 | 0.754 |
| 32 | $10^{-3}$ | 0.977 | 0.863 | 0.797 | 0.747 |
| 64 | $10^{-5}$ | 0.980 | 0.864 | 0.799 | 0.741 |
| 64 | $10^{-4}$ | 0.980 | 0.868 | 0.805 | 0.745 |
| 64 | $10^{-3}$ | 0.979 | 0.866 | 0.797 | 0.745 |

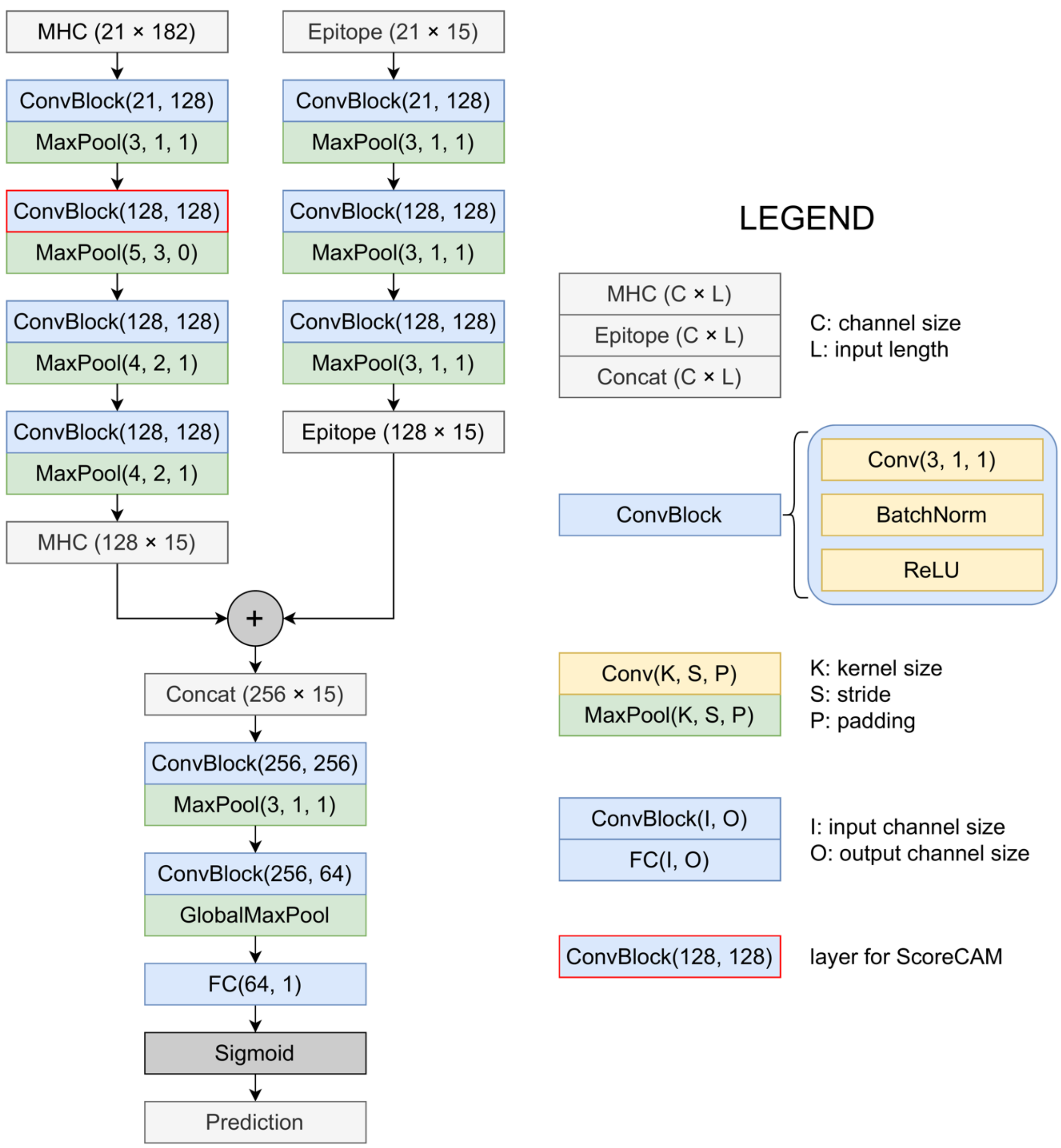


**Supplementary Figure 1. The CNN model architecture of MHCfovea's predictor.** The predictor adopted by MHCfovea is an ensemble model of multiple CNN model. Each CNN model takes both MHC-I sequence and epitope sequence as input. The MHC part and epitope part pass through four and three convolution blocks separately. Then, they are concatenated on the dimension of convolutional channels, and pass through another two convolution blocks followed by a max global pooling layer, a fully connective layer, and a sigmoid function to get the final prediction score. The convolution block with a red box is the layer for the ScoreCAM process.

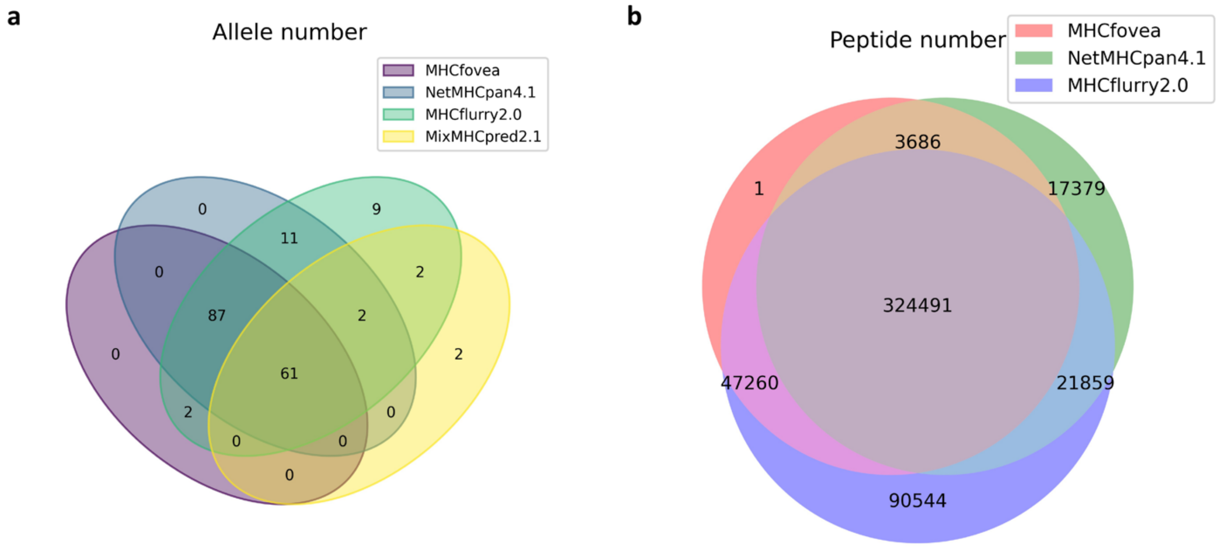


**Supplementary Figure 2. The training data comparison between predictors. a**, A Venn diagram of the sets of human MHC-I alleles in the training datasets of different predictors, including NetMHCpan4.1, MHCflurry2.0, MixMHCpred2.1, and MHCfovea. **b**, A Venn diagram of the sets of experimental measurements used in the training process of NetMHCpan4.1, MHCflurry2.0 and MHCfovea. Of note, for NetMHCpan4.1, multi-allelic data was removed because of multiple alleles.

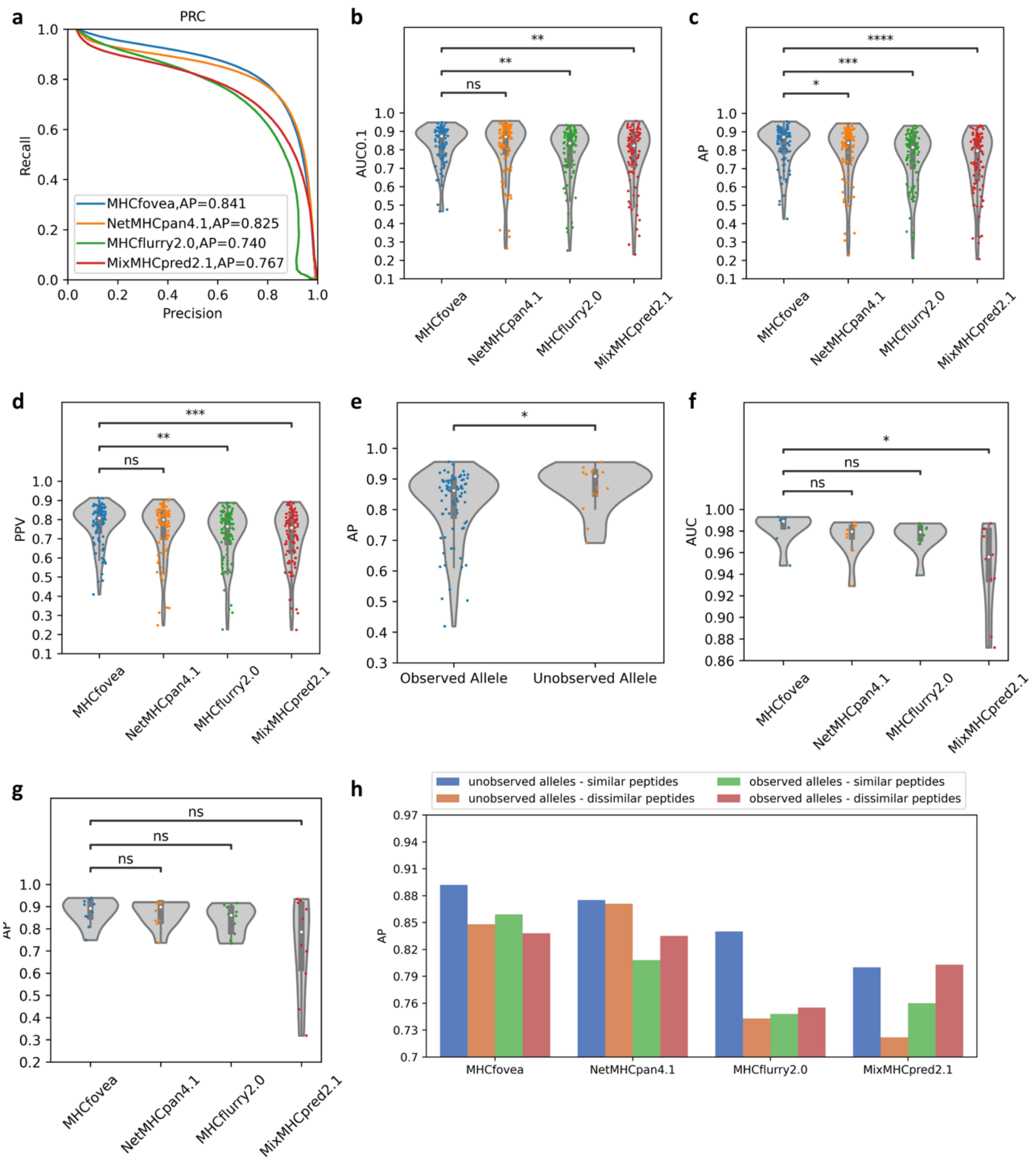


**Supplementary Figure 3. Performance of MHCfovea's predictor.** The following performances are all applied on the benchmark dataset. **a**, The precision-recall curves with AP depict the comparison between predictors. **b-d**, Violin plots depict the distribution of AUC0.1, AP, and PPV of each predictor by alleles (allele number=91, one allele was removed because it is unavailable in MixMHCpred2.1). **e**, Comparison of the AUC between observed (n=76) and unobserved (n=16) alleles. **f-g**, Violin plots depict the distribution of AUC and AP by commonly observed alleles of all the four predictors (allele number=10). **h**, The comparison of AP on the four groups split from the benchmark dataset between predictors. Violin plots depict the median value with a white dot, the 75th and 25th percentile upper and lower hinges, respectively, and whiskers with 1.5x interquartile ranges. P-values (two-tailed independent t-test) are shown as "ns" no significance, * $P \leq 0.05$, ** $P \leq 0.01$, *** $P \leq 0.001$, and **** $P \leq 0.0001$. Source data and details of the statistical analysis are provided in Supplementary Data 2, 3, and 4.

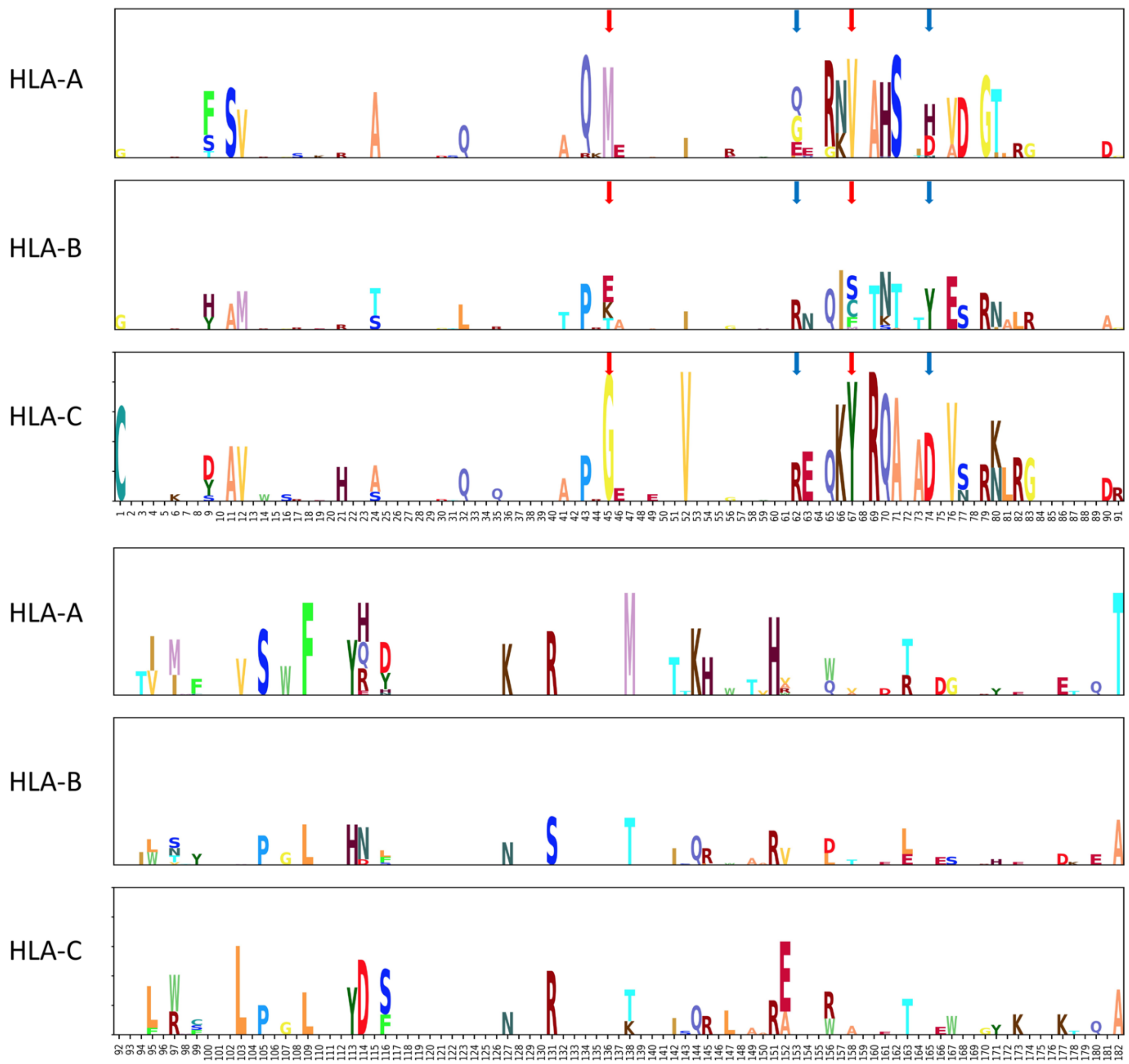


**Supplementary Figure 4. The sequence logo of MHC-I sequence derived from different HLA genes.** Only alleles from the training dataset are used to construct the position probability matrix (PPM). Each sequence logo is the positive part of difference between PPM of a specific HLA gene and PPM of all alleles. Each HLA gene has its own patterns on particular regions. Some positions are highly polymorphic for an HLA gene but conservative for others. For example, the positions annotated by red arrows are polymorphic in HLA-B but conservative for HLA-A and -C. In the same way, the positions annotated by blue arrows are polymorphic in HLA-A but conservative for HLA-B and -C.

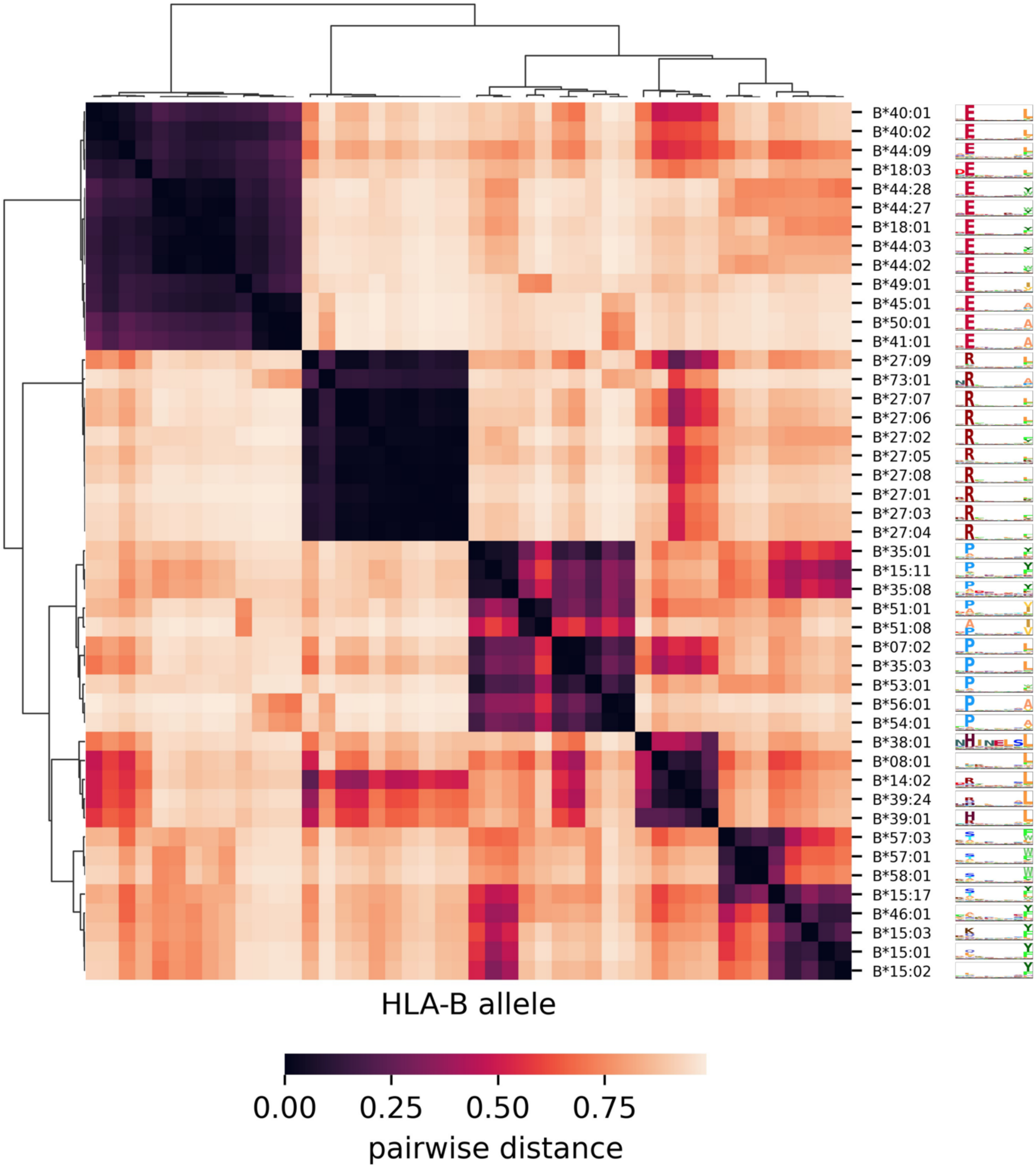


**Supplementary Figure 5. The heatmap clustering of MHC-I-binding motifs of HLA-B alleles in the training dataset.** A hierarchical clustering with cosine distance metric and UPGMA (unweighted pair group method with arithmetic mean) algorithm is used. In this figure, the clustering result was either dominated by the N-terminal or the C-terminal sub-motifs of the alleles. Alleles with similar N-terminal sub-motifs may have dissimilar C-terminal sub-motifs. For example, both HLA-B*07:02 and HLA-B*56:01 have a P-dominant N-terminal sub-motif, but the former has an L-dominant C-terminal sub-motif and the latter has an A-dominant one.

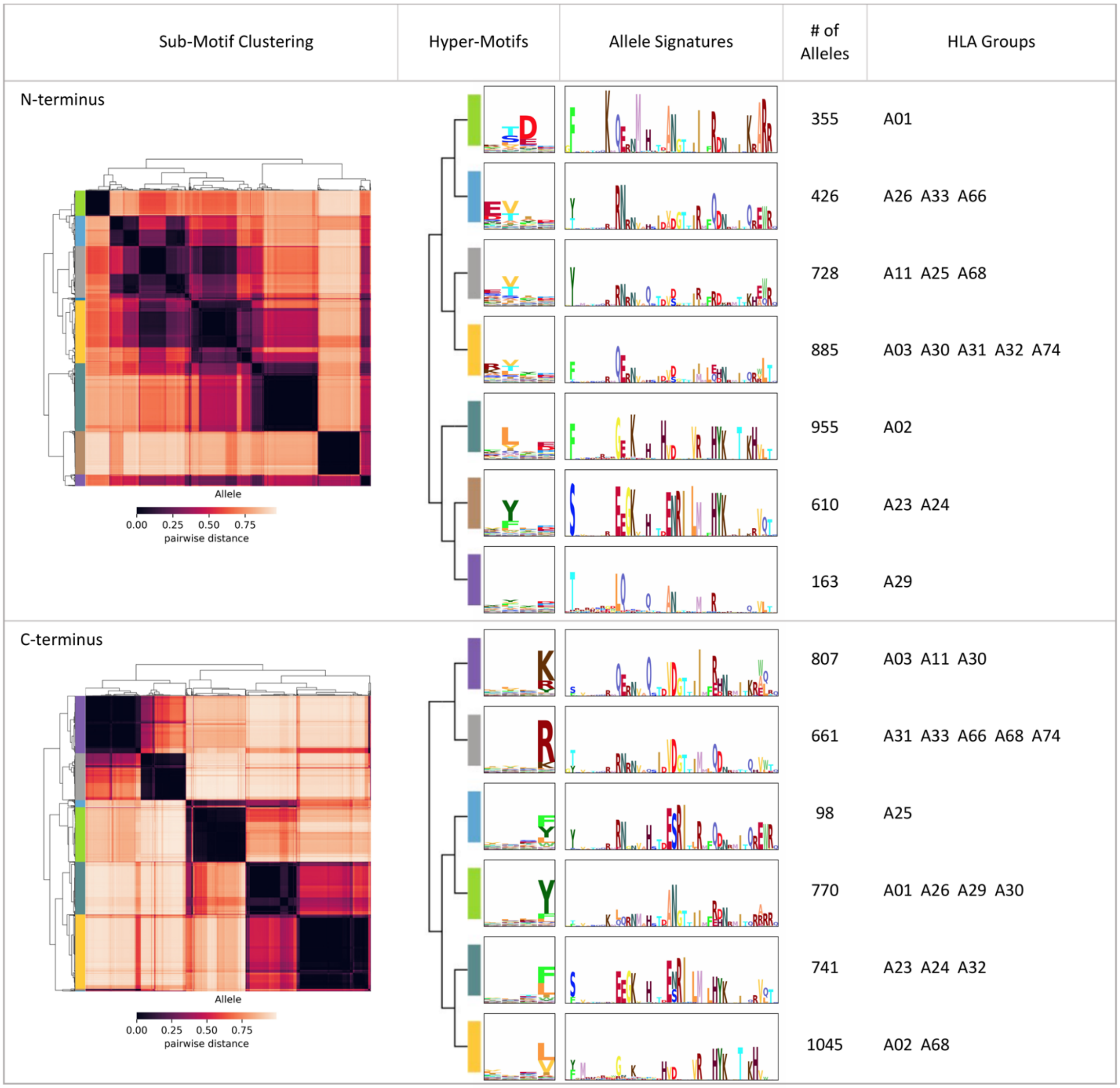


**Supplementary Figure 6. A summarization table of HLA-A.** The MHC-I-binding motifs are divided into N-terminal and C-terminal sub-motifs; sub-motifs are clustered by agglomerative hierarchical clustering. Hyper-motifs and the corresponding allele signatures are calculated for each sub-motif cluster. In each cluster, the number of alleles, and the HLA groups with the number of alleles ≥25, are recorded in the last two columns. Source data are provided in Supplementary Data 7.

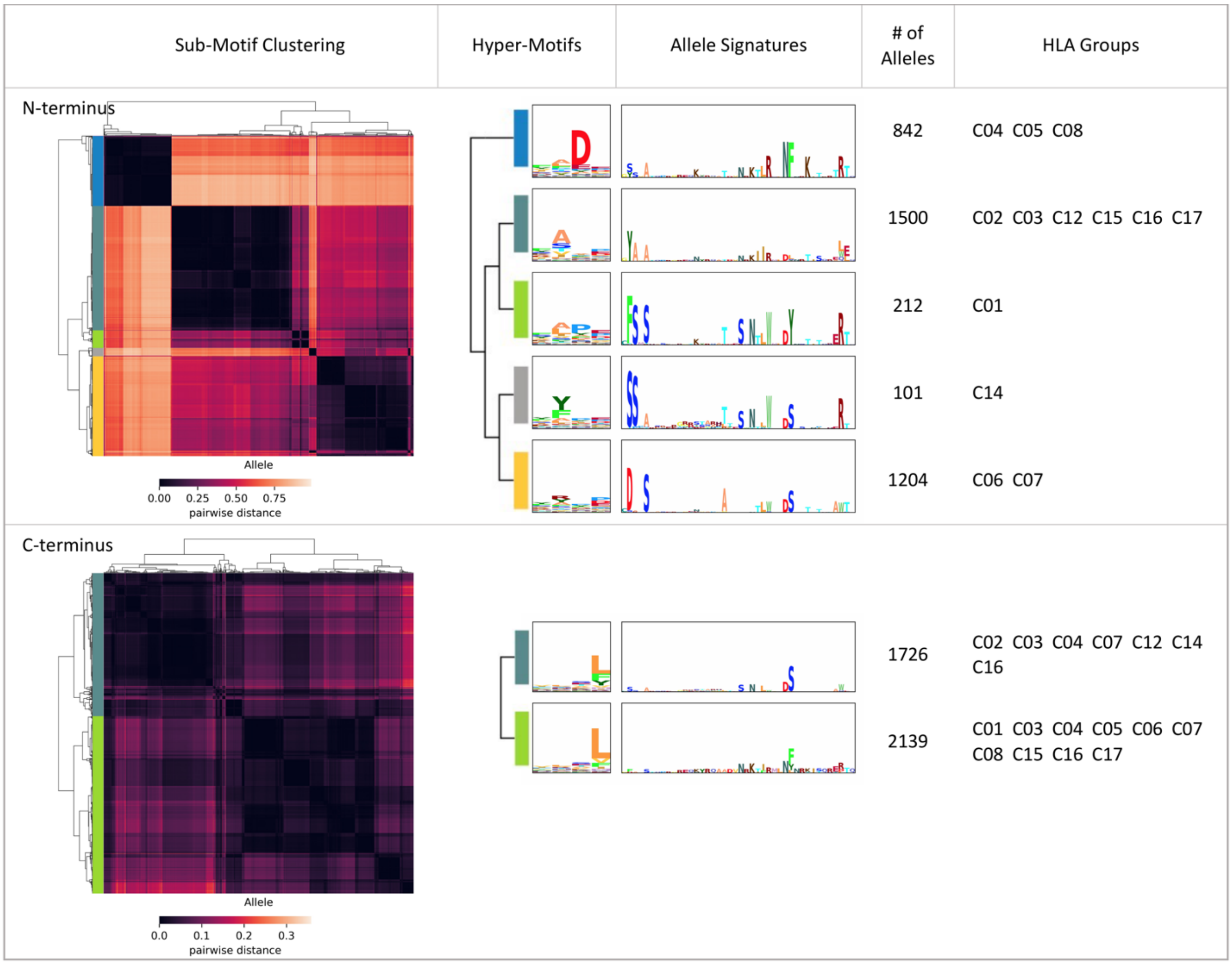


**Supplementary Figure 7. A summarization table of HLA-C.** The MHC-I-binding motifs are divided into N-terminal and C-terminal sub-motifs; sub-motifs are clustered by agglomerative hierarchical clustering. Hyper-motifs and the corresponding allele signatures are calculated for each sub-motif cluster. In each cluster, the number of alleles, and the HLA groups with the number of alleles ≥25, are recorded in the last two columns. Source data are provided in Supplementary Data 7.

a

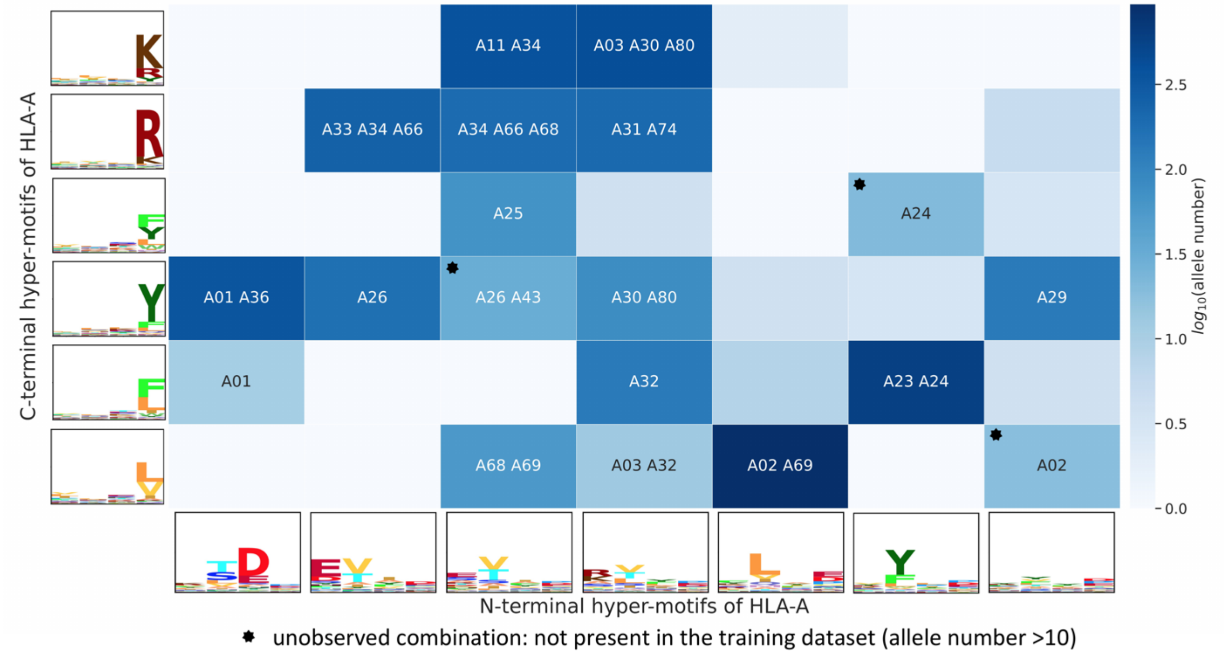


b

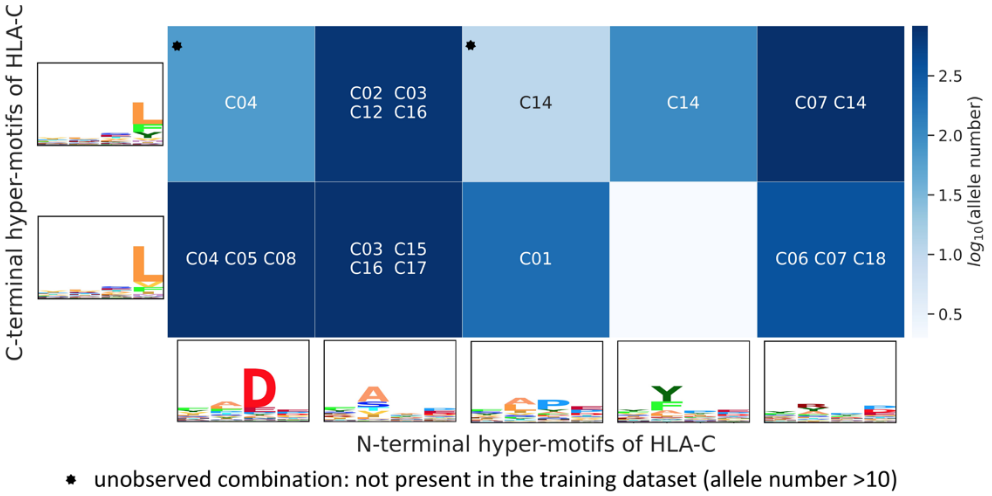


**Supplementary Figure 8. The combination map of N-terminal and C-terminal hyper-motifs. a-b**, The heatmap on the combinations of N-terminal (x-axis) and C-terminal (y-axis) hyper-motifs for HLA-A (**a**) and -C (**b**). The binding motif of an allele is a combination of an N-terminal and a C-terminal hyper-motif. After allocating all the alleles into the combination map, the cell color is determined by $\log_{10}$(number of alleles in the cell). In each cell with an allele number >10, the maximal HLA group, and HLA groups with an allele number ≥25, or with a proportion (the allele number in the cell to the overall number of an allele group) >0.1, are listed. Source data are provided in Supplementary Data 8.

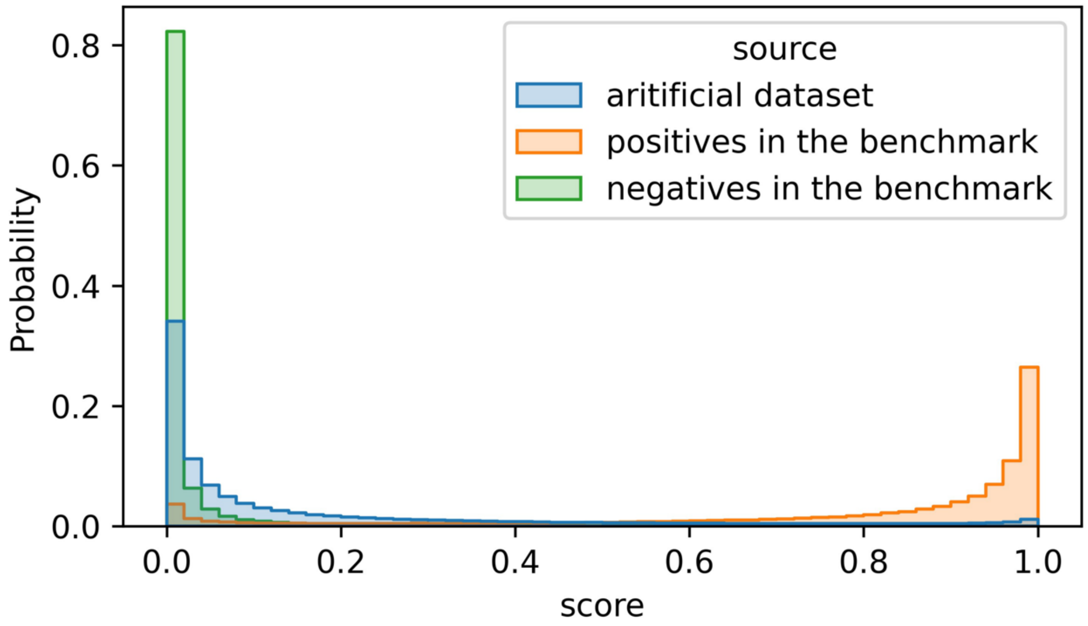


**Supplementary Figure 9. Analysis of the prediction on an artificial dataset (positive peptides in the training dataset, paired with all the alleles in the benchmark dataset).** The distribution of prediction scores on the three groups, including similar peptides paired with different alleles (the artificial dataset), the positive data in the benchmark, and the negative data in the benchmark.
