# Supplementary material for: Connecting MHC-I-binding motifs with HLA alleles via deep learning
Source: Commun Biol. 2021 Oct 18;4:1194. doi: 10.1038/s42003-021-02716-8 (PMC8523706; doi:10.1038/s42003-021-02716-8)
Supplement: Supplementary file 4 — Description of Supplementary Files [file 42003_2021_2716_MOESM4_ESM.docx]

**Description of Additional Supplementary Files**

**File Name:** Supplementary Data 1

**Description:** Data number of each dataset by alleles. The number of peptides by alleles from different sources including binding assay data, ligand elution data, and decoy data in the training, validation, and benchmark datasets.

**File Name:** Supplementary Data 2

**Description:** The data for Fig. 2d and Supplementary Fig. 3b-d. The performance of each predictor on the benchmark dataset by alleles.

**File Name:** Supplementary Data 3

**Description:** The data for Fig. 2e and Supplementary Fig. 3e-g. The performance of the MHCfovea’s predictor on the observed and unobserved alleles of the benchmark dataset, and the performance of each predictor on the common unobserved alleles of the benchmark dataset.

**File Name:** Supplementary Data 4

**Description:** The data for Fig. 2f and Supplementary 3h. Performance of the MHCfovea’s predictor and other predictors on the whole benchmark dataset, unobserved alleles, and dissimilar peptides.

**File Name:** Supplementary Data 5

**Description:** The data for Fig. 3a-b. The epitope mask and allele mask for the alleles in the training dataset.

**File Name:** Supplementary Data 6

**Description:** The data for Fig. 3b-d. The importance, polymorphism, and annotation of each position of MHC-I sequence.

**File Name:** Supplementary Data 7

**Description:** The data for Fig. 4 and Supplementary Fig. 6-7. The hyper-motif and allele signature of each cluster.

**File Name:** Supplementary Data 8

**Description:** The data for Fig. 5a and Supplementary Fig. 8. The cluster number of each allele.

**File Name:** Supplementary Data 9

**Description:** The data for Fig. 6a. The polymorphism on each position of each HLA group.

**File Name:** Supplementary Data 10

**Description:** The data for Fig. 6b. The performance of the MHCfovea’s predictor on the unobserved alleles within the mono-cluster or multi-cluster groups.
