# Supplementary material for: Connecting MHC-I-binding motifs with HLA alleles via deep learning
Source: Commun Biol. 2021 Oct 18;4:1194. doi: 10.1038/s42003-021-02716-8 (PMC8523706; doi:10.1038/s42003-021-02716-8)
Supplement: Supplementary file 15 — Reporting Summary [file 42003_2021_2716_MOESM15_ESM.pdf]

Corresponding author(s): Chien-Yu Chen

Last updated by author(s): Sep 11, 2021

Reporting Summary

Nature Research wishes to improve the reproducibility of the work that we publish. This form provides structure for consistency and transparency in reporting. For further information on Nature Research policies, see our Editorial Policies and the Editorial Policy Checklist.

Statistics

For all statistical analyses, confirm that the following items are present in the figure legend, table legend, main text, or Methods section.

n/a	Confirmed	
☐	☒	The exact sample size (*n*) for each experimental group/condition, given as a discrete number and unit of measurement
☒	☐	A statement on whether measurements were taken from distinct samples or whether the same sample was measured repeatedly
☐	☒	The statistical test(s) used AND whether they are one- or two-sided<br>*Only common tests should be described solely by name; describe more complex techniques in the Methods section.*
☒	☐	A description of all covariates tested
☒	☐	A description of any assumptions or corrections, such as tests of normality and adjustment for multiple comparisons
☐	☒	A full description of the statistical parameters including central tendency (e.g. means) or other basic estimates (e.g. regression coefficient) AND variation (e.g. standard deviation) or associated estimates of uncertainty (e.g. confidence intervals)
☐	☒	For null hypothesis testing, the test statistic (e.g. *F*, *t*, *r*) with confidence intervals, effect sizes, degrees of freedom and *P* value noted<br>*Give P values as exact values whenever suitable.*
☒	☐	For Bayesian analysis, information on the choice of priors and Markov chain Monte Carlo settings
☒	☐	For hierarchical and complex designs, identification of the appropriate level for tests and full reporting of outcomes
☐	☒	Estimates of effect sizes (e.g. Cohen's *d*, Pearson's *r*), indicating how they were calculated

Our web collection on statistics for biologists contains articles on many of the points above.

Software and code

Policy information about availability of computer code

Data collection	Data processing:<br>python (v3.6.9), numpy (v1.18.2), pandas (v1.0.3)<br><br>Developed Code:<br>All source codes are available at GitHub (https://github.com/kohanlee1995/MHCfovea) and Mendeley Data (http://dx.doi.org/10.17632/c249p8gdzd.3).
Data analysis	Data analysis:<br>python (v3.6.9), numpy (v1.18.2), pandas (v1.0.3), scikit-learn (v0.22.2)<br><br>Deep learning:<br>python (v3.6.9), pytorch (v1.4.0)<br><br>Visualization:<br>python (v3.6.9), matplotlib (v3.2.1), seaborn (v0.10.0), logomaker (v0.8)<br><br>Developed Code:<br>All source codes are available at GitHub (https://github.com/kohanlee1995/MHCfovea) and Mendeley Data (http://dx.doi.org/10.17632/c249p8gdzd.3).

For manuscripts utilizing custom algorithms or software that are central to the research but not yet described in published literature, software must be made available to editors and reviewers. We strongly encourage code deposition in a community repository (e.g. GitHub). See the Nature Research guidelines for submitting code & software for further information.

Data

Policy information about availability of data

All manuscripts must include a data availability statement. This statement should provide the following information, where applicable:

- Accession codes, unique identifiers, or web links for publicly available datasets
- A list of figures that have associated raw data
- A description of any restrictions on data availability

Several public databases were used in this study, including Immune Epitope Database and Analysis Resource (IEDB) (https://www.iedb.org/) for experimental measurements, UniProt (https://ftp.uniprot.org/pub/databases/uniprot/current_release/knowledgebase/complete/uniprot_sprot.fasta.gz) for decoy peptides, and IPD-IMGT/HLA (https://github.com/ANHIG/IMGTHLA/tree/3410) for MHC-I allele sequences.

Research data files supporting this study, including the peptide-binding cleft sequence of MHC-I alleles, the training, validation, and benchmark datasets, the prediction of the validation and benchmark datasets, and the prediction of the allele expansion are available from Mendeley Data (http://dx.doi.org/10.17632/c249p8gdzd.3). Source data for all figures are provided in Supplementary Data.

Website for the summarization of MHCfovea is available at https://mhcfovea.ailabs.tw

Field-specific reporting

Please select the one below that is the best fit for your research. If you are not sure, read the appropriate sections before making your selection.

☒ Life sciences ☐ Behavioural & social sciences ☐ Ecological, evolutionary & environmental sciences

For a reference copy of the document with all sections, see nature.com/documents/nr-reporting-summary-flat.pdf

Life sciences study design

All studies must disclose on these points even when the disclosure is negative.

Sample size	All experimental data of MHC-I binding and ligand elution assay available at IEDB were collected. Decoy peptides were extracted from the UniProt database. In the benchmark and validation dataset, the sample size of decoys is about 30 times larger than the size of eluted peptides. This ratio is close to that of the dataset in NetMHCpan4.1 [1]. In the training dataset, the sample size of decoys is about 90 times larger than that of eluted peptides. This ratio is three times larger than that of the validation dataset, which was decided via experiments in this study.<br><br>[1] Reynisson, B., Alvarez, B., Paul, S., Peters, B. & Nielsen, M. NetMHCpan-4.1 and NetMHCIIpan-4.0: improved predictions of MHC antigen presentation by concurrent motif deconvolution and integration of MS MHC eluted ligand data. Nucleic Acids Res. 48, 449–454 (2020).
Data exclusions	To focus on the prediction of 4-digit human MHC-I alleles (ex. A*01:01), non-human, mutant, and digital-insufficient MHC-I alleles were excluded. The peptides were restricted to 8-15-mers and this setting covered most epitopes [1].<br><br>[1] Trolle, T. et al. The Length Distribution of Class I–Restricted T Cell Epitopes Is Determined by Both Peptide Supply and MHC Allele–Specific Binding Preference. J. Immunol. 196, 1480–1487 (2016).
Replication	All experiments about prediction and analysis can be reproduced by cloning the GitHub repository (https://github.com/kohanlee1995/MHCfovea).
Randomization	No randomization was needed for building the dataset because we used the MHC-I immunopeptidome built by Sarkizova et al [1], which is the latest and largest mono-allelic mass spectrometry dataset, as the benchmark in this study.<br><br>[1] Sarkizova, S. et al. A large peptidome dataset improves HLA class I epitope prediction across most of the human population. Nat. Biotechnol. 38, 199–209 (2020).
Blinding	For a convincing evaluation of the model performance, the benchmark dataset was blinded to our predictor during the training process.

Reporting for specific materials, systems and methods

We require information from authors about some types of materials, experimental systems and methods used in many studies. Here, indicate whether each material, system or method listed is relevant to your study. If you are not sure if a list item applies to your research, read the appropriate section before selecting a response.

Materials & experimental systems

n/a	Involved in the study
☒	☐ Antibodies
☒	☐ Eukaryotic cell lines
☒	☐ Palaeontology and archaeology
☒	☐ Animals and other organisms
☒	☐ Human research participants
☒	☐ Clinical data
☒	☐ Dual use research of concern

Methods

n/a	Involved in the study
☒	☐ ChIP-seq
☒	☐ Flow cytometry
☒	☐ MRI-based neuroimaging
