# Supplementary material for: Connecting MHC-I-binding motifs with HLA alleles via deep learning
Source: Commun Biol. 2021 Oct 18;4:1194. doi: 10.1038/s42003-021-02716-8 (PMC8523706; doi:10.1038/s42003-021-02716-8)
Supplement: Supplementary file 3 — Editorial Assessment Report [file 42003_2021_2716_MOESM3_ESM.pdf]

nature portfolio

GUIDED OPEN ACCESS
nature methods | nature communications | communications biology

Editorial Assessment Report

Dear Professor Chen,

Thank you again for your patience during the review process and for choosing to submit your manuscript using the Guided Open Access pilot at the Nature Portfolio. As part of this process, our editorial team has considered your paper for three of our journals with strong interest in publishing in your field: *Nature Methods*, *Nature Communications*, and *Communications Biology*.

Your manuscript entitled "Connecting MHC-I-binding motifs with HLA alleles via deep learning" has now been reviewed by 2 experts in computational tool development, machine learning, biostatistics, and structural bioinformatics, whose comments are included in the attached Editorial Assessment Report. As part of the Guided Open Access pilot, editors from all 3 journals have discussed the reviewer reports and the manuscript's suitability for our journals. After careful evaluation, our editorial recommendation is to revise the manuscript and submit back through the Guided Open Access submission portal for consideration at *Communications Biology*.

In brief, for publication in *Communications Biology*, we would require you to address the referee concerns regarding methodological advance and benchmarking over related tools while using the same training or input data sets. You will find more details regarding the required revisions in the Editorial Assessment Report below. Please note that the Editorial Assessment Report is a standalone document that contains an editorial evaluation, recommendation and portable peer advice to help you navigate and interpret the reviewers' reports. It also provides guidance for adhering to best practice with regard to transparency and reproducibility, for example on the issue of sharing data. We have also included information about data accessibility and reproducibility, which we hope you will find useful.

Should you have any questions about the recommended journal or would like advice on the revisions, you can contact me directly and I will be happy to assist. We look forward to receiving the revised version of your manuscript.

Yours sincerely,

George Inglis
--
George Inglis
Associate Editor
Communications Biology

On behalf of the Guided OA editorial team

Contents of this Report

- **Manuscript overview**: details about your manuscript and the editorial team.
- **Manuscript assessment**: personalised recommendation from the editors.
- **About the editorial process**: an overview of the Guided Open Access process.
- **Annotated reviewer comments**: the referee reports with comments from the editors.
- **Open research evaluation**: advice for adhering to best reproducibility practices.

Manuscript overview			
**Manuscript number**<br>GUIDEDOA-21-00041	**Submission date**<br>14 February 2021	**Decision date**<br>15 April 2021	
**Title**	Connecting MHC-I-binding motifs with HLA alleles via deep learning	**Corresponding author**	Chien-Yu Chen<br>ORCID: 0000-0002-9875-0066
**Preprint information**	No preprint found	**Peer review type**	Single-blind
**Editorial Assessment Team**	**Primary editor: George Inglis**<br>**Home Journal:** *Communications Biology*, ORCID: 0000-0002-9069-5242<br>**Editorial team members:**<br>Ingrid Knarston, *Nature Communications*, ORCID: 0000-0002-0932-8649<br>Madhura Mukhopadhyay, *Nature Methods*, ORCID: 0000-0003-3907-3955		
**About your primary editor**	George received his PhD in Genetics and Molecular Biology from Emory University, where he studied mouse models of voltage-gated sodium channel dysfunction and epilepsy. He also has research experience in epigenomics and in vitro models of neuronal development. George joined the editorial team of *Communications Biology* in September 2020 and is based in the New York office.		

Manuscript assessment and recommendation	
**Editor's summary of the manuscript and overall assessment**	Here, the authors have developed MHCfovea, a deep learning tool that can predict MHC-I binding motifs. When comparing AUC values, MHCfovea significantly outperforms three related tools: NetMHCpan4.1 (which also relies on deep learning), MHCflurry2.0, and MixmHCpred2.1. One benefit of this study is that the authors have also used MHCfovea to predict potential binding motifs for ~13,000 alleles, and summarize their results in an accessible online database.<br><br>Both reviewers acknowledge that MHCfovea may present several advantages over existing tools, but raise concerns over the suitability of the training and input data sets used for benchmarking. There were also concerns about whether ScoreCAM is appropriately integrated into MHCfovea. While we would ultimately require the complete code to be made publicly available at the time of publication, we believe it is critical for the training code to be provided to reviewers in a revised manuscript.<br><br>In summary, a resubmission should include, at a minimum, the following revisions:<br><br>(1) Benchmark the performance of MHCfovea to related tools using the same training data set from other studies (as suggested by Referee #1) and update training and input data sets (as suggested by Referee #2).<br><br>(2) Elaborate on the limitations of MHCfovea compared to other tools, as well as the lack of biological validation.<br><br>(3) Justify the inclusion and suitability of ScoreCAM to MHCfovea, or remove it from the analytical pipeline, as mentioned by both reviewers.<br><br>(4) Provide training code for reviewer evaluation, as mentioned by Referee #2.

Editorial assessment overview

Nature Methods	**Not feasible to meet the editorial requirements**	The technical advance (compared to prior literature) represented by MHCfovea is not sufficient for publication in *Nature Methods*.
Nature Communications	**Not feasible to meet the editorial requirements**	The technical advance (compared to prior literature) represented by MHCfovea is not sufficient for publication in *Nature Communications*.
Communications Biology	**Major revisions needed**	As noted by the reviewers, improved benchmarking (with appropriate data sets) and a qualified discussion of MHCfovea are required for publication.

**Editor's recommendation**	***Option 1*: Revise for consideration at Communications Biology**<br>While many revisions are needed, these do not require expansion of the work beyond proper benchmarking of the predictive ability of the approach compared to other tools. See the annotated reviewer comments for details.<br><br>***Option 2*: Revise for publication elsewhere**<br>While *Communications Biology* would be especially interested in considering your revised manuscript, we can share the reviews with another journal outside of the Nature Portfolio if requested.
**Next steps**	If you would like to follow our recommendation, when you are ready you can upload the revised manuscript, along with your point-by-point response to the reviewer's reports and editorial advice here*.

About the editorial process

By selecting the Nature Portfolio Guided Open Access option, your manuscript was assessed for suitability in three of our titles that provide venues for publication of high-quality work across the spectrum of methods development research: *Nature Methods, Nature Communications*, and *Communications Biology*. For more information about Guided Open Access, please see here.

Collaborative editorial assessment

Your editorial team discussed the manuscript to determine its suitability for the Nature Portfolio Guided OA pilot. Our assessment of your manuscript takes into account several factors, including whether the work meets the **technical standard** of the Nature Portfolio and whether the findings are of **immediate significance** to the readership of at least one of the participating journals in the Nature Portfolio Guided Open Access methods cluster.

Peer review

Experts were asked to evaluate the following aspects of your manuscript:

Novelty in comparison to prior publications;

Likely audience of researchers in terms of broad fields of study and size;

Potential impact of the study on the immediate or wider research field;

Evidence for the claims and whether additional experiments or analyses could feasibly strengthen the evidence;

Methodological detail and whether the manuscript is reproducible as written;

Appropriateness of the literature review.

Editorial evaluation of reviews

Your editorial team discussed the potential suitability of your manuscript for each of the participating journals. They then discussed the revisions necessary in order for the work to be published, keeping each journal's specific editorial criteria in mind.

Journals in the Nature portfolio will support authors wishing to transfer their reviews and (where reviewers agree) the reviewers' identities to journals outside of Springer Nature. For any questions about review portability, please contact our editorial office: guidedoa@nature.com

Annotated Reviewer Reports

The editor has included some additional comments on the specific points raised by the reviewers below. However, please note that all points should be addressed in a revision, even if the editor has not specifically commented on them.

Reviewer #1

Report received: 19 March 2021

**Reviewer #1**	This reviewer has not chosen to waive anonymity. The reviewer's identity can only be shared with representatives of an established journal editorial office.
**Reviewer #1 expertise** *Summarised by the editor*	This reviewer has expertise in statistical genomics and computational tool development.
**Editor's comments about this review**	This reviewer raises excellent points on how to appropriately benchmark MHCfovea to other tools (particularly in points #1, 4, and 11), while also offering useful advice on how to improve figure presentation (see point #7) and the applicability of this tool (point #2). In a separate conversation with the editor, this reviewer also expressed concerns regarding the lack of biological validation of results from MHCfovea. While we agree that the study would benefit from biological validation or interrogation of predicted motifs from MHCfovea, this point would not be necessary for publication at *Communications Biology* and instead could be addressed as a limitation.
**Reviewer #1 comments**	
**Overview**	Lee et al. present a novel method, MHCfovea, for predicting peptide-binding by MHC molecules and inferring peptide motifs and MHC allele signatures using deep learning.<br><br>MHCfovea addressed the imbalanced classification of MHC-peptide binding prediction by taking advantage of the ensemble strategy with down-sampling. In the evaluation with independent data of eluted ligand, MHCfovea outperformed other methods. MHCfovea detected important positions in MHC sequences using ScoreCAM. They detected multiple binding motifs, and inferred meaningful hyper-motifs with allelic signatures by clustering binding motifs.<br><br>The manuscript is well written and convincing the value of their method. However, there are several points that should be addressed.
**Specific comments**	

#	Reviewer comment	Editorial comment
**Major concerns for revision**		
**1**	The improvement of prediction accuracy in prediction of peptide-binding is valuable. Could you describe more about whether the improvement is due to just the difference in a dataset used in training or any advantages in the algorithm? Which previous methods were trained on the current data set? If the improvement is probably due to just the difference in datasets, it should be noted as such.	**It would be critical to confirm that MHCfovea outperforms related tools regardless of the training data set.**
**2**	The idea of outputting probability scores is good; however, I wonder if there is a downside to not outputting %rank, which is commonly used in other tools. What is the practical threshold for the score that shows significant bindings, and is it validated?	**If feasible, please include %rank as an output of MHCfovea. At a minimum, the use of probability scores should be fully justified for consideration at *Communications Biology*.**
**3**	It is interesting to evaluate the relationship between the degree of polymorphism and the sensitivity maps derived from ScoreCAM. However, I am not familiar with ScoreCAM, but I slightly wonder if the gradient is likely to be large where many categorical variables can be taken due to the original nature of the algorithm.	**This comment is also relevant to point #11 from Referee #2. A revised manuscript should either further justify the inclusion of ScoreCAM, or remove it entirely from the MHCfovea pipeline.**
**4**	The method of tuning hyper-parameters of the deep learning model should be written. Which data were used and what optimization method was used?	**For the sake of reproducibility, it would be important to clearly state training data sets and optimization methods.**
**Minor concerns for revision**		
**5**	In page 8, the first sentence of the third paragraph of “Selection of important MHC-I residues”, I cannot understand why “non-polymorphic” positions were chosen as important positions. Isn’t it “polymorphic”?	
**6**	In page 9 and 10, the third sentence of the first paragraph and the fourth sentence of the third paragraph, the knowledge that the second and last positions of residues are important might be general; however, should be supported by citing a literature.	
**7**	Some of the figures may be difficult to understand because of missing labels and legends (e.g. horizontal axis of Fig. 3a, dot legend of Fig. 3b (a round dot is all	**Please update Figures 3/5 with additional definitions and graphical keys, as suggested. In**

	residues?), and axis and heatmap label of Fig 5a).	**general, we would recommend uploading individual image files with a revised manuscript to improve figure resolution.**<br><br>**Please note that our formatting style ultimately requires the legend to clearly define all abbreviations, symbols, colors, and shading. Please write out the symbols/colors in words (blue circles, red dashed line, etc.) within these definitions.**
**8**	Some abbreviation should be decoded the first time they are mentioned in the main text (e.g. AUC, PR).	
**Comments on impact, strength of the claims, and reproducibility**		
**9**	**Impact of the study.** The implementation of the algorithm is good, but the idea may not be fully novel. A study of predicting peptide-binding of MHC molecules using deep learning is now commonplace	**The fact that this area has been explored before with machine learning methods reduces the impact and enthusiasm for publishing in *Nature Communications*. Altogether, the novelty and impact is not sufficient for consideration at *Nature Methods*. However, *Communications Biology* believes MHCfovea would represent an advance over other algorithms, if additional benchmarking is included.**
**10**	**Strength of the claims.** The novelty in this work seems to be (1) the improvement in accuracy probably due to the ensemble learning; (2) the detection of important positions of MHC molecules visualization of the trained model, and binding-motif exploration.	
**11**	**Reproducibility.** To strengthen reproducibility, in performance comparisons with the previous methods, using the same input data would be warranted.	**As mentioned in Point #1, it would be essential to confirm that MHCfovea outperforms related tools regardless of the training data set, for consideration in *Communications Biology*.**

		**The level of performance advance once this retraining is factored in (also highlighted by Reviewer #2) was concerning for *Nature Communications*.**

**Reviewer #2** *Report received:* 30 March 2021	
**Reviewer #2**	This reviewer has not chosen to waive anonymity. The reviewer's identity can only be shared with representatives of an established journal editorial office.
**Reviewer #2 expertise** *Summarised by the editor*	This reviewer has expertise in machine learning, proteomics, and structural bioinformatics.
**Editor's comments about this review**	This reviewer offers a useful perspective to validate MHCfovea performance using relevant training and input data sets. In particular, we believe that addressing points #6-7 would be essential for *Communications Biology* to consider a revised manuscript.
**Reviewer #2 comments**	
**Overview**	This work introduces MHCfovea, a deep Neural Network (NN) to predict the interaction between couples of MHC-I alleles and peptides. Along the prediction process, MHCfovea also shows which amino acids in the MHC-I target are deemed of interest in the interaction. This information can be used to build allele- and peptide-specific signatures through unsupervised learning (clustering).<br><br>The text clearly states the objectives of this study and is overall well written. The code, available on github, is well organised and easy to use for evaluation (the training code is not available). The performance of the predictor seems to exceed the state of the art.
**Specific comments**	

#	Reviewer comment	Editorial comment
**1**	**Impact:** Although this is not the first NN-based MHC-I binding predictor, it seems to me like it is the first to include elements from deep learning frameworks that can be used to explain the predictor's choices and learn new information about the underlying biological process (i.e. which amino acids play a role in the binding). This seems like a good idea, especially in a moment where	

	explainability in AI is very much an issue.<br><br>However, I have to point out some important flaws in the methods of this study that undermine the credibility of its results and have to be addressed before the study can be published.	
**Major comments for revision.**		
**2**	The main issue is the way the train, validation and benchmark sets are built. Although the authors state in the text that no allele/peptide couple in the benchmark set is identical to any in the train/validation sets, I am not convinced that this is enough to avoid information leakage between the sets and, subsequently, to an overestimation of the performance of the predictor. Each NN in the ensemble is made of approximately 500k weights, while the training set is made of approximately of 260k positive (binding) samples. A NN of such size could easily overfit the positive data so that these samples are "memorized" in it, which would make it unreliable when presented with previously unseen alleles or peptides. Special care has to be taken, then, that no samples are too similar not only between training and benchmark sets, but also between training and validation sets.	**These issues related to training and biases within the datasets and consequent impact on model performance were an editorial concern for *Nature Communications*.**
**3**	In the case of the validation set, here the samples are a random 5% of the total training set. This means that samples in the validation set might be near-identical to those in the training set, making it hard to judge if the NN is overfitting the data at training time, since it will also implicitly overfit similar samples in the validation set.	
**4**	In the case of the benchmark set, even though no two allele/peptide couples are identical with the training set, all alleles (except one) in the benchmark set are also used in the training/validation sets. Upon closer inspection of the data that was made available by the authors on Mendeley, I have also seen that for a 1000 random peptides from the "positive" samples in the benchmark set ("test.csv"), 353 of those were identical to "positive" peptides in the training set "train_hit.csv" and 429 where near-identical (i.e. the benchmark peptides were a sub-sequence of the training peptides). When doing the same test on "negative" peptides from the benchmark set, only six of those were identical to any "positive" peptide in the	

	training set. It is not hard to imagine, then, that such an oversized NN might just recall identical or near-identical peptides (or near-identical allele/peptide couples) from the training set and still perform well on the "unseen" benchmark.	
**5**	This also makes me doubt that MHCfovea's good performance on rare alleles is trustworthy enough that we can use the NN to expand its usage to the larger set of 13k alleles, since an overfitting NN will usually give unexpected results on unseen data.	
**6**	In order to ensure that MHCfovea's performance is not result of overfitting, I suggest the authors to re-train and test it on new datasets so that:<br>a. No peptides are identical or near-identical (e.g. one is a subsequence of the other) between training/validation set and benchmark set<br>b. No peptides are identical or near-identical between training set and validation set<br>c. No allele in the train set is identical to those in the validation set<br>d. No allele in the train set is identical to those in the benchmark	**MHCfovea analysis should be repeated following these criteria, for consideration at *Communications Biology*.**
**7**	While I realize that points 6c and 6d might excessively limit the size of the train and/or benchmark sets, it would be possible to perform testing so that different versions of the NN are trained on different, non-overlapping subsets of train/benchmark data (see cross-fold or leave-one-out validation).	
**Minor comments for revision:**		
**8**	Could the authors explain how they settled on this particular NN architecture? Have other architectures been explored, such as RNNs (which usually perform better when dealing with AA sequences)?	
**9**	Why is the final activation of the NN a sigmoid and not a softmax, since this looks like a classification problem (prediction of binding/non-binding instead of, say, binding affinity)?	
**10**	Related to the previous point: it seems to me like it would be incorrect to say that MHCfovea predicts binding probability, since the last activation is not a softmax, and	

	the final prediction is the average of 18 different predictors. Please address or fix this.	
**11**	Judging by the ScoreCAM paper, it seems like it should not be possible to apply it as is on MHCfovea (ScoreCAM is made for classification networks, and MHCfovea is not). Could the authors better specify how ScoreCAM was adapted to this particular architecture?	**This comment echoes point #3 from Referee #2; the use of ScoreCAM should be justified in a revised manuscript.**
**12**	Please describe more in detail how the training set is split in downsized datasets (line 100 and following) since this was not immediately clear to me	
**13**	Please state explicitly that the best ensemble was selected based on validation data (and not, say, on test data). This point is implied in the text but I think it would be best to say it clearly	
**14**	Line 185: “the allele signature was constructed from a subset of alleles in the cluster”, perhaps I missed this from somewhere else in the text, but how is this subset constructed?	
**15**	Line 118: “less” should be “fewer”	
**16**	Line 174: “meaning” should be “meaningful”	
**17**	**Reproducibility:** The evaluation code and full datasets have been made available by the authors and I was able to reproduce some of the results on this work. The training code is not available, so this work is not fully reproducible (i.e. generating similar NN models from the training data as well as the CAM scores)	**Please make this code immediately available for reviewer input. We would recommend adding this information to your current GitHub page.**

Open Research Evaluation	
**Data Availability**	
**Data Availability Statement**	This journal strongly supports public availability of data and custom code associated with the paper in a persistent repository where they can be freely and enduringly accessed or as a supplementary data file when no appropriate repository is available. If data and code can only be shared on request, please explain why in your data Availability Statement, and also in the correspondence with your editor. For more information, please refer to https://www.nature.com/nature-research/editorial-policies/reporting-standards#availability-of-data<br><br>Please ensure that datasets deposited in public repositories are now publicly accessible, and that accession codes or DOI are provided in the "Data Availability" section. As long as these datasets are not public, we cannot proceed with the acceptance of your paper. For data that have been obtained from publicly available sources, please provide a URL and the specific data product name in the data availability statement. Data with a DOI should be further cited in the methods reference section.<br><br>See our policy page for details.
**Mandatory data deposition**	http://www.nature.com/authors/policies/availability.html#data
**Source data**	The following figure panels should be accompanied by the underlying source data: **Fig. 2b-g, Fig. 3a-b, Fig. 6a-b, Supp. Fig. 2.**<br><br>Source data files (in Excel or text format) will be mandatory prior to publication in a Nature Portfolio journal.
**Data citation**	Where datasets are hosted in public repositories that provide datasets with Digital Object Identifiers (DOIs), we encourage

	these datasets to be formally cited in reference lists. Citations of datasets, when they appear in the reference list, should include the minimum information recommended by DataCite and follow journal style. For example:<br><br>Hao, Z., AghaKouchak, A., Nakhjiri, N., Farahmand, A. Global Integrated Drought Monitoring and Prediction System (GIDMaPS) Data sets. *figshare*. http://dx.doi.org/10.6084/m9.figshare.853801 (2014)<br><br>Citing and referencing data in publications supports reproducible research, by increasing the transparency and provenance tracking of data generated or analysed during research. Citing data formally in reference lists also helps facilitate the tracking of data reuse and may help assign credit for individuals' contributions to research. A number of Springer Nature imprints are signatories of the Joint Declaration on Data Citation Principles, which stress the importance of data resources in scientific communication.
**Data Publication Recommended**	We suggest that you consider publishing your MHCfovea dataset as a Data Descriptor in *Scientific Data*. See the journal website for details.<br><br>https://www.nature.com/sdata/
**Code Availability**	
**Code Availability**	As stated by Referee #2 (point #17), please provide training code for reviewer input. We would recommend adding this code to the existing Github page for MHCfovea.<br><br>See our policy page for details.
**Code Citation**	In addition to making the custom code available, we ask that you ensure that the version of the code/software described in the paper is deposited in a DOI-minting repository (eg, Zenodo) and that this DOI is also cited in the main Reference list. See here for details.
**Code Reporting**	Please ensure that any software mentioned in the Reporting

	Summary (Python, numpy, etc.) are also listed in the manuscript.
**Research ethics**	
**Research with human participants**	Research involving human research participants must have been performed in accordance with the Declaration of Helsinki . Please provide confirmation that all relevant ethical regulations were followed and that informed consent was obtained. This must be stated in the Methods section, including the name of the board and institution that approved the study protocol. See our ethics policy for details.<br><br>**Please ensure that relevant ethics statements from source data sets are included in the revised manuscript.**
**Methods assessment and reproducibility**	
**Data Presentation**	• Please ensure that data presented in a plot, chart or other visual representation format shows data distribution clearly (e.g. dot plots, box-and-whisker plots). When using bar charts, please overlay the corresponding data points (as dot plots) whenever possible and always for n ≤ 10. (Please see the following editorial for the rationale behind this request and an example https://www.nature.com/articles/s41551-017-0079).<br><br>• Large datasets must be supplied as separate Supplementary Data files. Each file must be labelled as Supplementary Data 1, etc. **Please reclassify Supplementary Tables 2, 3, and 5 as Supplementary Data 1-3.**
**Statistical Evaluation**	• Wherever statistics have been derived (e.g. error bars, box plots, statistical significance) the legend needs to provide and define the n number (i.e. the sample size used to derive statistics) as a precise value (not a range), using the wording “n=X biologically independent

	samples/animals/cells/independent experiments/n= X cells examined over Y independent experiments" etc. as applicable.<br><br>• Please note that statistics such as error bars significance and p values cannot be derived from n<3 and must be removed in all such cases.<br><br>• We strongly discourage deriving statistics from technical replicates, unless there is a clear scientific justification for why providing this information is important. Conflating technical and biological variability, e.g., by pooling technically replicates samples across independent experiments is strongly discouraged. (For examples of expected description of statistics in figure legends, please see the following https://www.nature.com/articles/s41467-019-11636-5 or https://www.nature.com/articles/s41467-019-11510-4).
**Figure Legends**	• The figure legends must indicate the statistical test used. Where appropriate, please indicate in the figure legends whether the statistical tests were one-sided or two-sided and whether adjustments were made for multiple comparisons.<br><br>• For null hypothesis testing, please indicate the test statistic (e.g. F, t, r) with confidence intervals, effect sizes, degrees of freedom and P values noted.<br><br>• Please provide the test results (e.g. P values) as exact values whenever possible and with confidence intervals noted. **Please update the legends of Figures 2e-2g, 6a-6b, and Supplementary Figures 2a-2f, 2h, accordingly.**<br><br>• All error bars need to be defined in the legends (e.g. SD, SEM) together with a measure of centre (e.g. mean, median). For example, the legends should state something along the lines of "Data are presented as mean values +/- SEM" as appropriate.<br><br>• All box plots need to be defined in the legends in

	terms of minima, maxima, centre, bounds of box and whiskers and percentile. **Please update Fig. 6, accordingly.**
**Reproducibility**	Please state in the legends how many times each experiment was repeated independently with similar results. This is needed for all experiments, but is particularly important wherever results from representative experiments (such as micrographs) are shown. If space in the legends is limiting, this information can be included in a section titled "Statistics and Reproducibility" in the methods section.
**Other notes**	
**Attached Reporting Summary**	We have included as an attachment to the decision letter a version of your Reporting Summary with a few notes. This is mainly for your information, but we hope it is helpful when preparing your revised manuscript. If you decide to resubmit the manuscript for further consideration at *Communications Biology*, please be sure to include an updated Reporting Summary.
